# Development and Assessment of a Novel Palmitoylation-Related lncRNA Signature for Prognosis and Immune Landscape in Hepatocellular Carcinoma

**DOI:** 10.32604/or.2025.070567

**Published:** 2026-01-19

**Authors:** Zhilong He, Jing Qin, Sixuan Wu, Xian Liang, Yu Liu, Jinfeng Qiu, Zhimin Li, Kai Hu

**Affiliations:** 1 Department of Radiation Oncology, The First Affiliated Hospital of Guangxi Medical University, Nanning, 530021, China; 2 Department of Oncology, The First Affiliated Hospital, Hengyang Medical School, University of South China, Hengyang, 421001, China

**Keywords:** Palmitoylation, long non-coding RNAs, hepatocellular carcinoma, prognosis

## Abstract

Objective: The contribution of long non-coding RNAs (lncRNAs) associated with protein palmitoylation to the progression of hepatocellular carcinoma (HCC) remains largely unclear. This study sought to establish a prognostic signature based on palmitoylation-related lncRNAs and explore their functional implications in HCC. Methods: RNA sequencing and clinical data for HCC and normal tissues were sourced from the Cancer Genome Atlas (TCGA). Pearson correlation analysis was used to identify lncRNAs that were co-expressed with palmitoylation-related genes. Univariate Cox regression was applied to select lncRNAs with prognostic value, followed by the construction of a predictive model using the least absolute shrinkage and selection operator (LASSO) regression. A focused analysis was performed on one key lncRNA, AC009403.1. Expression levels of the final nine lncRNAs included in the model were further validated by reverse transcription quantitative polymerase chain reaction (RT-qPCR). Results: A prognostic model for HCC was developed using nine palmitoylation-associated lncRNAs: AC009403.1, AC010789.1, AC026402.2, AC107021.2, AC135050.6, AL353572.4, MKLN1-AS, PRRT3-AS1, and ZNF582-AS1. This model effectively stratified patients into high- and low-risk groups exhibiting significantly different overall survival (OS) and progression-free survival (PFS), with the low-risk group showing more favorable outcomes. The high-risk group was associated with an immunosuppressive microenvironment, higher tumor mutation burden (TMB), and increased sensitivity to certain chemotherapeutic drugs (e.g., Sorafenib). Finally, RT-qPCR validation revealed that all nine lncRNAs were significantly upregulated in HCC tissues. Conclusion: The nine-lncRNA signature exhibits robust predictive power for HCC prognosis and provides novel insights into the mechanisms of lncRNA-regulated palmitoylation in HCC development.

## 1 Introduction

Liver cancer ranks as the sixth most commonly diagnosed cancer and the third leading cause of cancer-related mortality globally, with approximately 905,677 new cases and 830,180 deaths estimated in 2022 [1]. HCC represents the most prevalent form of primary liver cancer, accounting for 75%–85% of all cases [2]. The current mainstays of treatment for HCC include liver transplantation, surgical resection, percutaneous ablation, radiotherapy, and both transarterial and systemic therapies. From biological and immunological perspectives, HCC patients typically have a low to moderate tumor mutation burden (TMB), with an average of 2–9 mutations per megabase, corresponding to approximately 40–60 somatic coding mutations for which targeted therapies are currently lacking. The prognosis of HCC is heavily influenced by tumor characteristics, including tumor burden, vascular infiltration, and differentiation [3]. Additionally, the presence of liver cancer stem cells (LCSCs) and tumor-associated macrophages (TAMs) contributes significantly to hepatocarcinogenesis, drug resistance, metastasis, and recurrence [4]. To advance toward precision medicine in HCC, there is a pressing need to identify reliable biomarkers that can guide prognostic assessment and therapeutic decisions across all disease stages. Although survival rates have improved to some extent, the majority of HCC cases are diagnosed at advanced stages, leading to poor outcomes for many patients. Therefore, a deeper understanding of the pathogenesis of HCC and the identification of novel, reliable screening methods are crucial for developing promising strategies for cancer prevention and treatment.

Palmitoylation is a reversible post-translational modification where a 16-carbon palmitate molecule is covalently attached to specific cysteine residues on target proteins. This process, catalyzed by zinc finger DHHC-type containing (ZDHHC) enzymes, enhances the hydrophobicity of specific protein domains, thereby influencing their subcellular localization, stability, and protein-protein interactions [5,6]. Notably, these enzymes have been implicated in various diseases, including neurological disorders such as Huntington’s disease, schizophrenia, and intellectual disabilities [7,8], as well as autoimmune diseases and cancers [9,10]. Palmitoylation regulates a wide array of proteins, including oncogenes and tumor suppressors, and aberrant expression of ZDHHC proteins has been closely linked to the initiation and progression of several cancers [11], such as those of the lung, kidney, and liver [12–14], significantly impacting patient treatment responses and prognosis.

Long non-coding RNAs (lncRNAs) are a diverse group of non-coding RNA molecules longer than 200 nucleotides [15]. They modulate various molecular mechanisms, including chromatin remodeling [16,17], gene transcription [18], and translation [19]. Dysregulated expression of lncRNAs is frequently associated with the regulation of genes involved in metabolism, cell death, angiogenesis, and cancer metastasis [20]. Interestingly, some lncRNAs have the ability to encode small peptides from open reading frames, which have been implicated in cancer development [21]. LncRNAs also play a critical role in HCC; when abnormally expressed, they can interact with DNA, RNA, or proteins, or encode small peptides, thereby influencing cancer hallmarks [22]. Moreover, emerging evidence indicates that lncRNAs can regulate palmitoylation-mediated modifications. For instance, the knockdown of the lncRNA DUXAP8 has been demonstrated to impede the palmitoylation of the protein SLC7A11, thereby affecting its localisation at the cell membrane. Additionally, the lncRNA ZBTB10 has been shown to promote androgen receptor function by inducing S-palmitoylation [23,24]. However, the relationship between palmitoylation-associated lncRNAs and HCC remains unclear.

In this study, we employed a comprehensive bioinformatics approach to analyze data from the Cancer Genome Atlas (TCGA) database. Our methods included correlation analysis, prognostic model construction, functional enrichment, as well as assessments of tumor microenvironment, TMB, and drug sensitivity, followed by experimental validation. We aimed to develop a robust prognostic signature based on palmitoylation-related lncRNAs, ultimately evaluating its potential clinical utility for diagnosis, tailoring therapeutic strategies, and improving outcome prediction in HCC. Notably, lncRNA AC009403.1 was significantly overexpressed in HCC and constituted a core component of our prognostic signature. Its functional role in HCC, however, remains entirely unexplored. We therefore hypothesized that a novel prognostic signature based on palmitoylation-associated lncRNAs could effectively stratify HCC patients and predict clinical outcomes. Furthermore, we proposed that the signature, particularly its core component AC009403.1, might not only serve as an independent prognostic biomarker but also reflect the immunosuppressive tumor microenvironment and differential drug sensitivity, potentially offering a valuable tool for guiding personalized therapeutic strategies.

## 2 Materials and Methods

### 2.1 Data Acquisition

Data were obtained from TCGA (https://www.cancer.gov/tcga, accessed on 25 September 2025), which provides RNA expression, somatic mutation data, and clinical characterization for HCC. We downloaded transcriptomic data and clinicopathological characteristics, including age, sex, tumor stage, and histologic grade, for 424 subjects, comprising 374 tumor and 50 adjacent non-tumor samples. Samples lacking essential clinical features, such as overall survival time, American Joint Committee on Cancer (AJCC) TNM stage, or key demographic variables (e.g., age and sex), were excluded. This filtering resulted in a final cohort of 370 samples with complete clinical annotations for subsequent analysis.

### 2.2 Correlation Analysis and the Creation of Risk Scores

A Wilcoxon rank-sum test was employed to compare gene expression between normal and HCC tissues (*p* < 0.05). Next, Pearson correlation analysis was performed to identify lncRNAs co-expressed with palmitoylation-related genes, using stringent criteria of a correlation coefficient |R| > 0.4 and *p* < 0.001. Univariate Cox regression analysis was then conducted on these lncRNAs, integrating gene expression data with clinical survival information to screen for those with prognostic potential. To refine the signature and prevent overfitting, a LASSO Cox regression analysis was applied to the candidate lncRNAs derived from the univariate analysis. The optimal penalty parameter (λ) was determined through 10-fold cross-validation based on the ‘lambda.1se’ criterion, which selects the most parsimonious model. This analysis was conducted using the ‘glmnet’ package (version 4.1.10) in R. Subsequently, all eligible HCC patients were randomly assigned in a 1:1 ratio to a training set and a test set. The training cohort was used to construct the risk score model. Based on the results of the LASSO Cox regression, a prognostic risk score was calculated for each patient. The equation was as follows:

$$\text{RiskScore}=\sum_{i=1}^{n}\;\left({\text{Expression}}_{i}\times {\text{Coefficient}}_{i}\right)$$

where *n* is the number of prognostic lncRNAs, and the subscript *i* represents each individual lncRNA in the signature.

Based on the median risk score calculated within each cohort, patients in both cohorts were stratified into low-risk and high-risk groups. Survival analysis was performed to compare outcomes between the groups. The predictive accuracy of the risk score model was assessed using time-dependent receiver operating characteristic (ROC) curve analysis [25].

### 2.3 Creation and Validation of Predictive Diagrams

A nomogram was constructed using the R package ‘rms’ (version 8.0.0) to visualize the prognostic model, incorporating independent clinical risk factors and the calculated risk score. The performance of the nomogram was evaluated by assessing its calibration, which compares predicted probabilities with observed outcomes. A calibration curve was plotted to visually evaluate the agreement between the predictions of the nomogram and the actual survival rates.

### 2.4 Functional Enrichment Analysis

Differentially expressed genes related to palmitoylation were identified using the R ‘limma’ package (version 3.64.1). Kyoto Encyclopedia of Genes and Genomes (KEGG) enrichment analysis was conducted to identify molecular signaling pathways [26]. Gene Ontology (GO) enrichment analysis was used to classify and characterize gene and protein activities [27]. Gene Set Enrichment Analysis (GSEA) was performed to compare functional and pathway differences between the two groups [28].

### 2.5 Risk Score, Tumor Microenvironment, and TMB Assessment

The ESTIMATE algorithm was employed to calculate the stromal and immune scores between the two patient groups. Somatic mutations in HCC patients were analyzed using the R package ‘maftools’ (version 2.24.0), which processes Mutation Annotation Format (MAF) files [29]. Additionally, the relationship between TMB and the two risk groups was evaluated.

### 2.6 Drug Sensitivity Analysis

Drug sensitivity was assessed using the R ‘oncoPredict’ package (version 1.2), which determined the IC_50_ values of commonly used HCC treatment drugs [30]. The primary aim of this analysis was to evaluate drug response in the two patient groups.

### 2.7 Cell Culture and Reverse Transcription Quantitative Polymerase Chain Reaction (RT-qPCR)

HCC cell lines HuH7 (iCell-h080), SNU-449 (iCell-h502), and MHCC-97H (iCell-h143), as well as the normal human hepatocyte cell line THLE-2 (iCell-h388), were procured from iCell Bioscience Inc. (Shanghai, China). All cell lines were routinely tested and confirmed to be free of mycoplasma contamination and were authenticated by Short Tandem Repeat (STR) profiling. Cells were thawed in a 37°C water bath and then cultured in 10-cm dishes with Dulbecco’s Modified Eagle Medium (DMEM; Gibco, Thermo Fisher Scientific, cat. no. 11965092, Waltham, MA, USA) supplemented with 10% fetal bovine serum (FBS; Gibco, Thermo Fisher Scientific, cat. no. A5670701, Waltham, MA, USA) and 1% penicillin/streptomycin (Gibco, Thermo Fisher Scientific, cat. no. 15140122, Waltham, MA, USA). Cells were incubated at 37°C in a humidified 5% CO2 atmosphere. RNA was extracted from these cell lines using TRIzol reagent (Invitrogen, Thermo Fisher Scientific, cat. no. 15596026, Waltham, MA, USA), followed by cDNA synthesis with the PrimeScript RT kit (Servicebio, cat. no. G3329-100, Wuhan, China). Quantitative PCR (qPCR) was performed using the SYBR Green-based master mix (Servicebio, cat. no. G3326-05, Wuhan, China). Each 20 μL reaction mixture contained 10 μL of SYBR Green master mix, 2 μL of cDNA template, 0.8 μL of each forward and reverse primer, and 6.4 μL of nuclease-free water. The thermocycling protocol consisted of an initial denaturation at 95°C for 30 s, followed by 40 cycles of 95°C for 15 s and 60°C for 30 s. Melting curve analysis was conducted to verify amplification specificity. GAPDH was used as an internal reference gene for normalization, and the relative gene expression was calculated using the 2^-ΔΔCt^ method. Primer sequences are provided in Supplementary Table S1.

### 2.8 Statistical Analysis

Data analysis and visualization were conducted using R software (version 4.2.2) and GraphPad Prism 9.0 (GraphPad Software, LLC, San Diego, CA, USA). Based on the TCGA cohort comprising 370 HCC patients, survival differences between the high- and low-risk groups were compared using the Kaplan-Meier method with a two-sided log-rank test, and the predictive accuracy of the risk score model was evaluated by time-dependent receiver operating characteristic (ROC) curve analysis, presented as the area under the curve (AUC). The Wilcoxon rank-sum test was applied to analyze gene expression differences between tumor and normal tissues from the TCGA database. Principal component analysis (PCA) was performed to visualize the overall distribution and clustering of samples based on different gene sets. *In vitro* data derived from at least three independent biological replicates are presented as the mean ± standard deviation. Statistical comparisons between two groups were performed using Student’s *t*-test, while comparisons among multiple groups were conducted using one-way analysis of variance (ANOVA) followed by an appropriate post-hoc test. The false discovery rate (FDR) correction was applied for multiple testing where necessary. Statistical significance was defined as a two-sided *p* < 0.05 or an FDR-adjusted *p* < 0.05.

## 3 Results

### 3.1 Construction and Validation of an HCC Prognostic Model Based on Palmitoylation-Related lncRNAs

**Figure 1:**
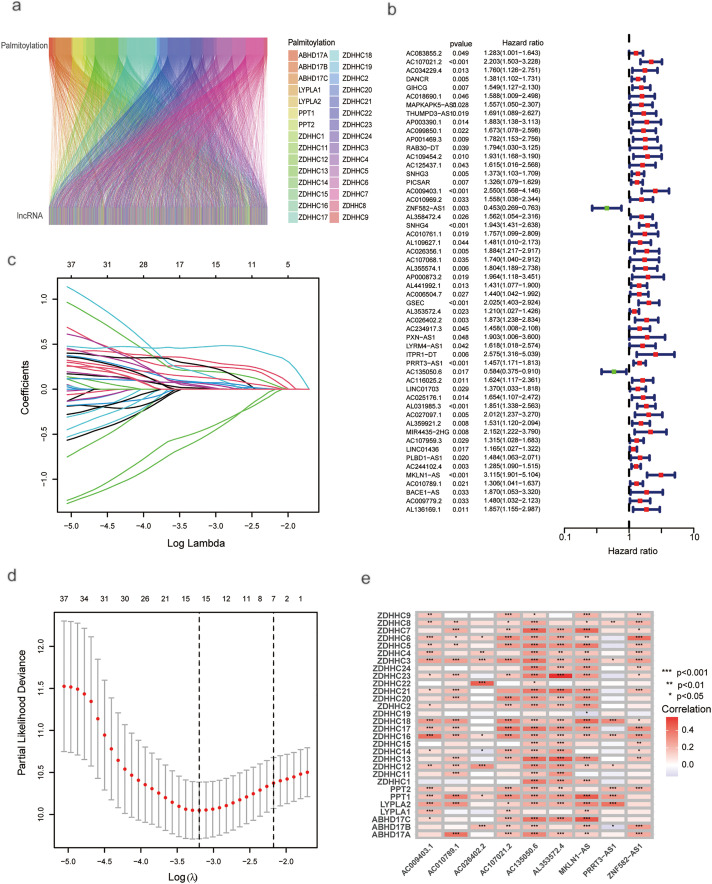
Univariate and LASSO analysis of lncRNAs associated with prognosis in HCC patients. (**a**) ZDHHC-related lncRNAs. (**b**) Univariate Cox regression analysis for screening prognostic lncRNAs associated with ZDHHC. (**c**) LASSO regression. (**d**) Profiles of LASSO coefficients. (**e**) Nine palmitoylation-associated lncRNAs linked to liver cancer prognosis (*p < 0.05; **p < 0.01; ***p < 0.001)

A total of 722 lncRNAs correlated with palmitoylation-related genes were identified, based on correlation coefficients > 0.4 and *p*-values < 0.001 (Fig. 1a). Complete correlation results for all palmitoylation-related gene-lncRNA pairs are provided in Supplementary Table S2. The expression matrix of these palmitoylation-associated lncRNAs is provided in Supplementary Table S3. Univariate Cox regression screened 54 palmitoylation-associated lncRNAs that significantly affected the survival of HCC patients (Fig. 1b). LASSO regression analysis, incorporating survival time, status, and lncRNA expression, identified a risk prediction model consisting of nine palmitoylation-related lncRNAs (AC009403.1, AC010789.1, AC026402.2, AC107021.2, AC135050.6, AL353572.4, MKLN1-AS, PRRT3-AS1, ZNF582-AS1) at the optimal λ (Fig. 1c–e). The risk score for each patient was calculated based on the expression levels and correlation coefficients of the nine lncRNAs from the LASSO analysis. The formula is as follows: risk score = (AC009403.1 × 0.5040) + (AC107021.2 × 0.3993) + (ZNF582-AS1 × −0.8379) + (AL353572.4 × 0.1949) + (AC026402.2 × 0.5477) + (PRRT3-AS1 × 0.2828) + (AC135050.6 × −1.0010) + (MKLN1-AS × 0.6704) + (AC010789.1 × 0.2690). The full list of lncRNAs with their corresponding coefficients is provided in Supplementary Table S4. Additional details regarding expression patterns, prognostic values, and known biological functions of these lncRNAs are summarized in Supplementary Table S5.

**Figure 2:**
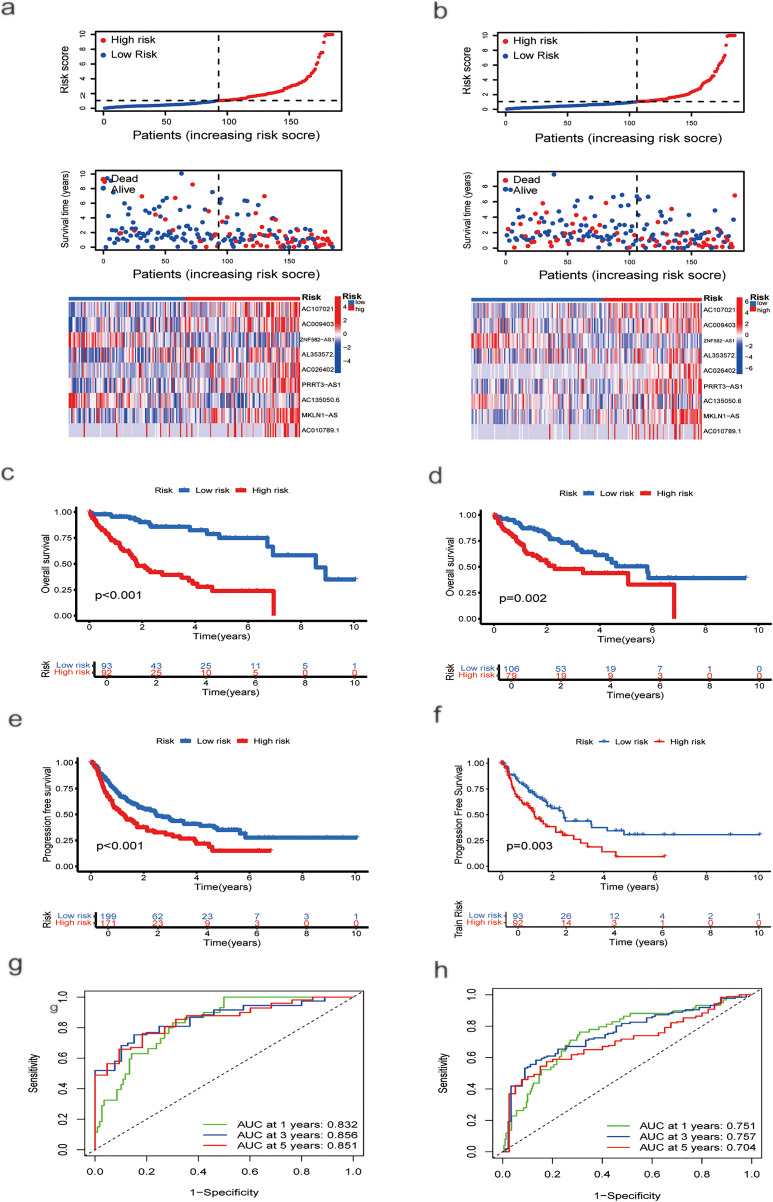
Risk Score Assessment and Categorization. (**a**) Distribution of nine prognostic lncRNAs in the training group based on risk score, survival status, and expression levels. (**b**) Distribution of the same genes in the testing group, based on risk score, survival status, and expression levels. (**c**) OS curve for HCC patients with different risk scores in the training group. (**d**) OS curve for HCC patients with different risk scores in the testing group. (**e**) PFS curves for HCC patients with different risk scores in the training group. (**f**) PFS curves for HCC patients with different risk scores in the testing group. (**g**) ROC curve analysis to assess the sensitivity and specificity in the training group. (**h**) ROC curve analysis to evaluate the sensitivity and specificity in the testing group

The cohort of 370 HCC patients was randomly split into training and testing sets of 185 patients each. Using the training set, a prognostic model was developed. As risk scores increased, the risk distribution plot indicated shorter survival times and higher recurrence rates (Fig. 2a). Kaplan–Meier analysis confirmed that patients in the low-risk group had significantly longer overall survival (OS) and progression-free survival (PFS) than those in the high-risk group (*p* < 0.001; Fig. 2c,e). The ROC curve demonstrated that the model achieved area under the curve (AUCs) of 0.832 (95% confidence interval (CI): 0.778–0.886), 0.856 (95% CI: 0.805–0.906), and 0.851 (95% CI: 0.800–0.902) at 1-, 3-, and 5-year survival, respectively (Fig. 2g). The model was subsequently validated in the test set. Using the same risk score formula and cutoff, patients were stratified into low- and high-risk groups. The risk distribution plot consistently showed increased mortality and recurrence associated with higher risk scores (Fig. 2b). The low-risk group exhibited significantly better OS (*p* = 0.002; Fig. 2d) and PFS (*p* = 0.003; Fig. 2f) than the high-risk group. The ROC curves for the test set yielded AUCs of 0.751 (95% CI: 0.689–0.813), 0.757 (95% CI: 0.695–0.819), and 0.704 (95% CI: 0.638–0.770) for 1-, 3-, and 5-year survival, respectively (Fig. 2h).

### 3.2 Construction of A Nomogram and Principal Component Analysis (PCA)

**Figure 3:**
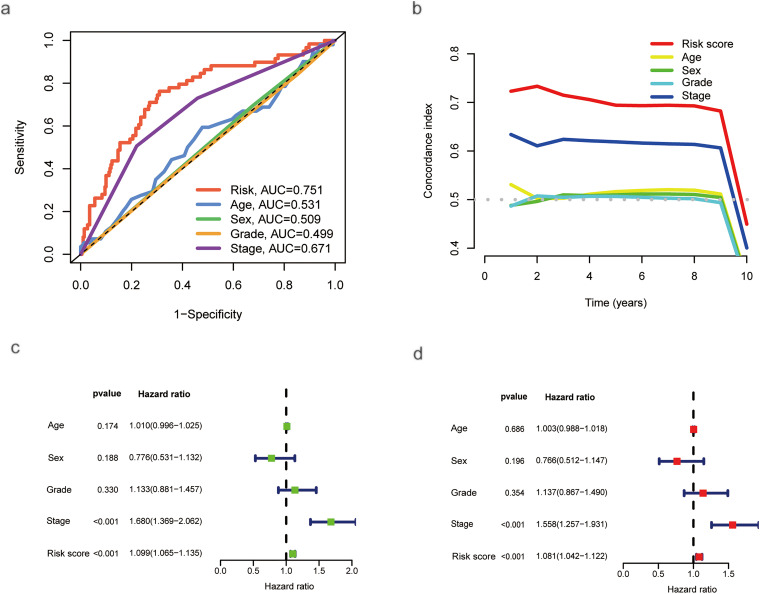

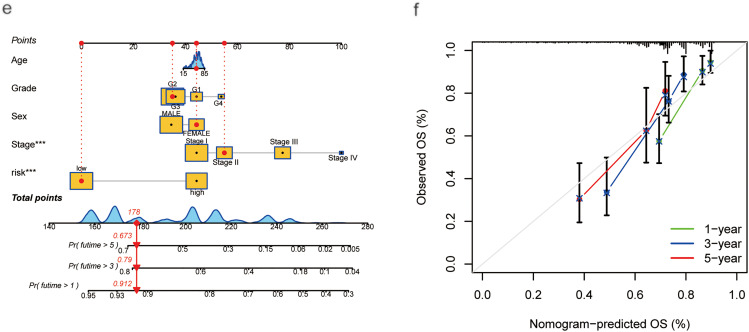
ROC Curves, COX regressions, and nomogram results based on risk scores and clinical characteristics. (**a**) ROC curve based on risk score, age, sex, grade, and stage. (**b**) C-index based on risk score, age, sex, grade, and stage. (**c**) Univariate Cox regression analysis of the effects of clinical characteristics and risk scores on HCC prognosis. (**d**) Multivariate Cox regression analysis of the effects of clinical characteristics and risk scores on HCC prognosis. (**e**) Prediction of OS in HCC patients based on clinical characteristics and risk scores using a nomogram. (**f**) Calibration curve showing the accuracy of the nomogram’s survival predictions (***p < 0.001)

**Figure A1:**
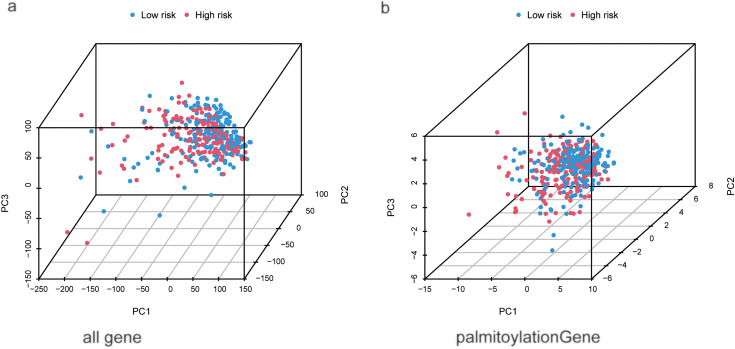

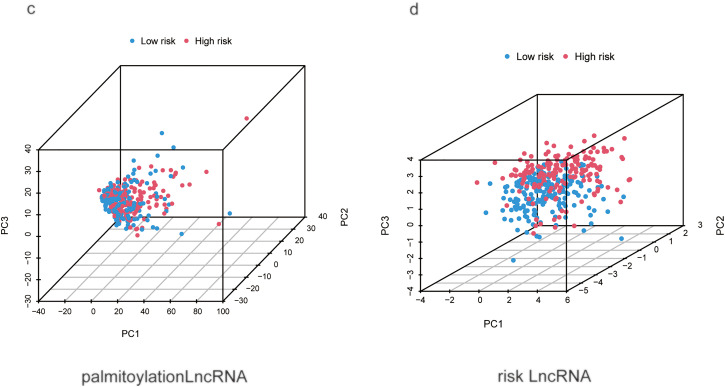
Principal component analysis (PCA) of different genomic features for distinguishing high- and low-risk HCC patients. (**a**) PCA of all genes. (**b**) PCA of palmitoylation-related genes. (**c**) PCA of all palmitoylation-associated lncRNAs. (**d**) PCA of the nine prognostic lncRNAs constituting the risk signature

Univariate analysis identified stage (hazard ratio (HR) = 1.680, 95% CI 1.369–2.062) and risk score (HR = 1.099, 95% CI 1.065–1.135) as significant risk factors for overall survival (Fig. 3c). Multivariate analysis, adjusted for age, sex, and grade, confirmed that both stage (HR = 1.558, 95% CI 1.257–1.931, *p* < 0.001) and the risk score (HR = 1.081, 95% CI 1.042–1.122, *p* < 0.001) remained independent prognostic factors (Fig. 3d). A nomogram was developed by integrating these independent predictors—stage and the risk score—along with other clinical variables (age, sex, grade) using multivariate Cox regression (Fig. 3e). The predictive performance of the risk score was superior to that of conventional clinical variables, achieving an AUC of 0.751 in the test set as shown in the ROC analysis (Fig. 3a). Model reliability was further corroborated by the concordance index (C-index; Fig. 3b) and calibration curves (Fig. 3f). To visualize the distribution of samples from the high- and low-risk groups, we conducted principal component analysis (PCA) based on four distinct sets of genomic features (Fig. A1). Specifically, PCA of all genes (Fig. A1a), palmitoylation-related genes (Fig. A1b), and all palmitoylation-associated lncRNAs (Fig. A1c) showed considerable overlap between the high- and low-risk groups. In contrast, PCA utilizing only the nine lncRNAs constituting the risk model showed a relatively clear visual separation of the two groups (Fig. A1d).

### 3.3 GO, KEGG, and GSEA Analysis

**Figure 4:**
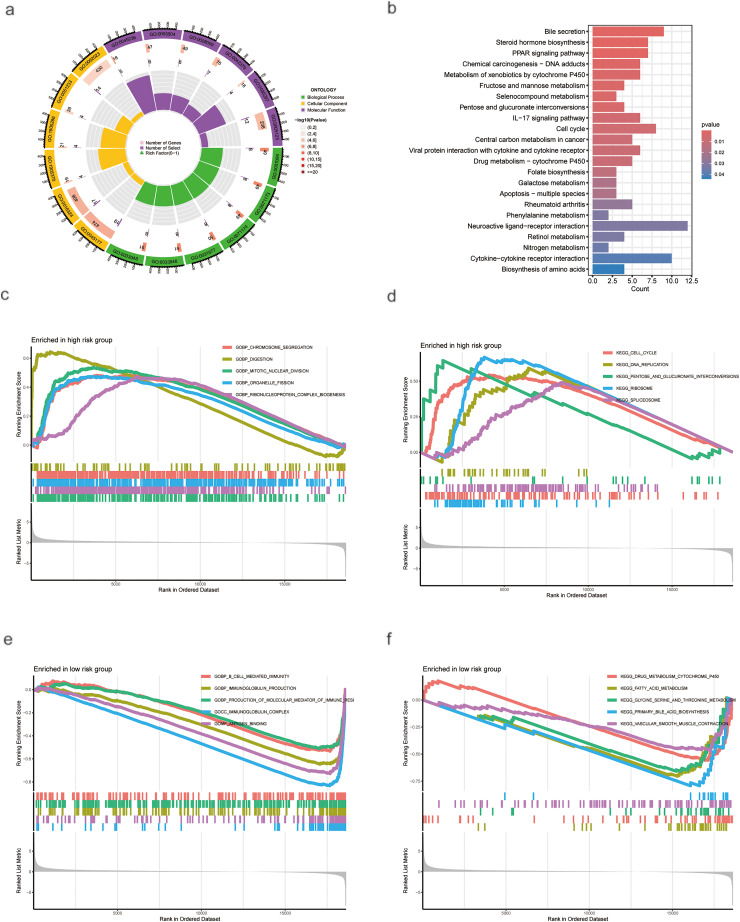
Enrichment and GSEA Analysis of Differentially Expressed Genes (DEGs). (**a**) GO analysis of DEGs. (**b**) KEGG analysis of DEGs. (**c**,**d**) Pathways are enriched in the high-risk group. (**e**,**f**) Pathways enriched in the low-risk group

GO and KEGG enrichment analyses indicated that differentially expressed genes were primarily enriched in biological processes, including bile secretion, the cell cycle, and the PPAR signaling pathway (Fig. 4a,b). GSEA further delineated distinct biological characteristics between the risk groups. The high-risk group was significantly enriched in cell cycle-related processes, including mitotic nuclear division, organelle fission, chromosome segregation, and ribonucleoprotein complex biogenesis (Fig. 4c). Consistent with this, KEGG pathway analysis also revealed that the high-risk group was associated with the regulation of cell cycle, DNA replication, ribosome, and spliceosome functions (Fig. 4d). Conversely, the low-risk group was predominantly related to immunomodulatory functions, such as immunoglobulin complex binding, antigen binding, immunoglobulin production, and B cell-mediated immunity (Fig. 4e). Additionally, both GSEA and KEGG analyses indicated that the low-risk group was significantly enriched in pathways governing cellular metabolism, including primary bile acid biosynthesis, fatty acid metabolism, and amino acid metabolism (Fig. 4f).

### 3.4 Association between Risk Score, Tumor Microenvironment, and TMB

**Figure 5:**
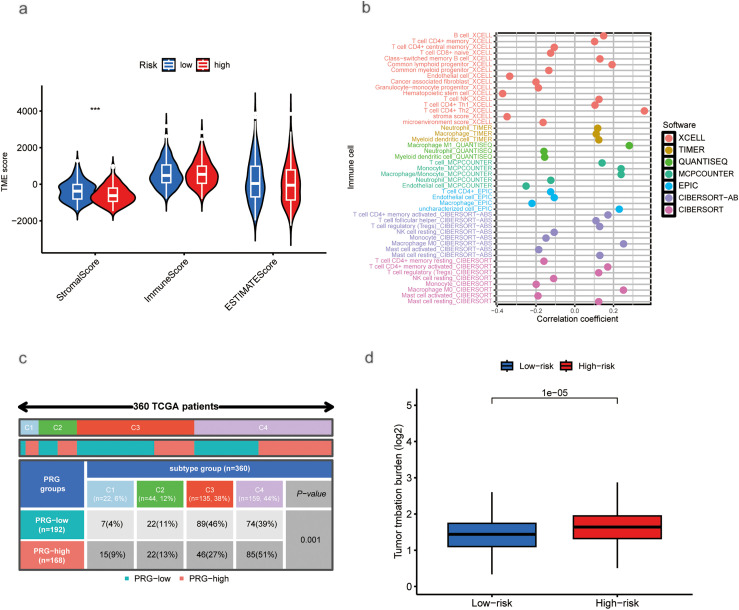

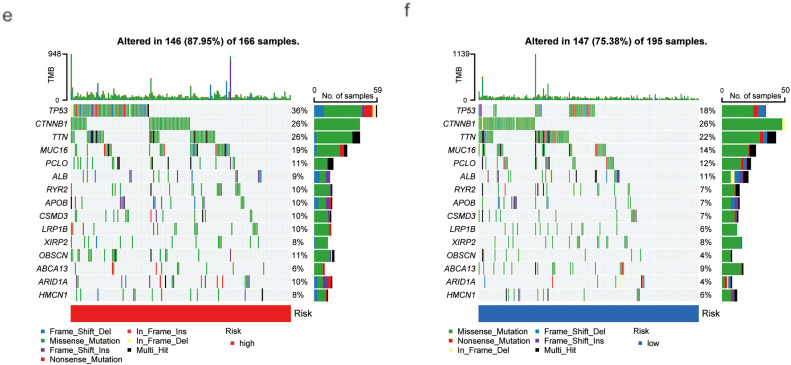
Association of Tumor Microenvironment (TME), Immune Cells, TMB, and Somatic Mutations with Risk Scores. (**a**) Correlation between stromal and immune cell scores in different risk groups. (**b**) Correlation between immune cell types and risk scores. (**c**) Immune typing analysis. (**d**) TMB expression in different risk groups. (**e**) Waterfall plots showing somatic mutations in the high-risk group. (**f**) Waterfall plots showing somatic mutations in the low-risk group (***p < 0.001)

Using the CIBERSORT algorithm, we assessed the relationship between risk scores and immune cell infiltration. Stromal scores were higher in the low-risk group compared to the high-risk group. In contrast, immune scores showed no significant difference between the two groups (Fig. 5a). Correlation analysis of immune cell types revealed a positive correlation between risk scores and various immune cells, including Th1 and Th2 CD4^+^ T cells, M0 and M1 macrophages, common lymphoid progenitors, activated memory CD4^+^ T cells, B cells, and regulatory T cells (Tregs). Conversely, negative correlations were found with activated mast cells, granulocyte-monocyte progenitors, neutrophils, resting memory CD4^+^ T cells, CD4^+^ T cells, CD8^+^ T cells, and resting NK cells (Fig. 5b). A significant disparity in immune subtype distribution was observed between high- and low-risk groups by the chi-square test (Fig. 5c). Mutation analysis of the TCGA-HCC cohort indicated that high-risk patients exhibited a higher TMB (*p* < 0.001; Fig. 5d). The most frequently mutated genes in high- and low-risk groups were TP53, CTNNB1, TTN, MUC16, PCLO, ALB, RYR2, APOB, and CSMD3 (Fig. 5e,f), with high-risk patients exhibiting notably higher mutation frequencies.

### 3.5 Drug Sensitivity Analysis Based on Risk Scores

**Figure 6:**
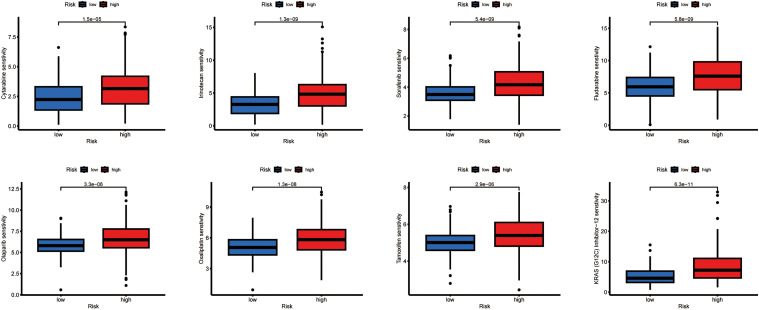

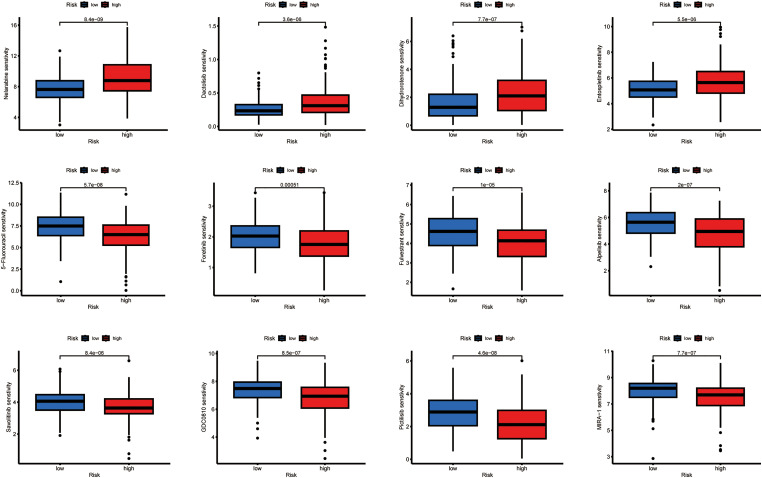
Relationship between drug sensitivity and different risk score groups. The exact *p*-value from the Wilcoxon rank-sum test is shown on the plot

Using computational estimation, we predicted differential responses to HCC therapeutics between the risk groups. The low-risk group was associated with heightened predicted sensitivity to agents including Cytarabine, Irinotecan, Sorafenib, Olaparib, Oxaliplatin, Fludarabine, Tamoxifen, and several other drugs, whereas the high-risk group exhibited a potential increased sensitivity to 5-Fluorouracil, Foretinib, Fulvestrant, and other agents (Fig. 6).

### 3.6 Individual lncRNA Analysis (AC009403.1)

**Figure 7:**
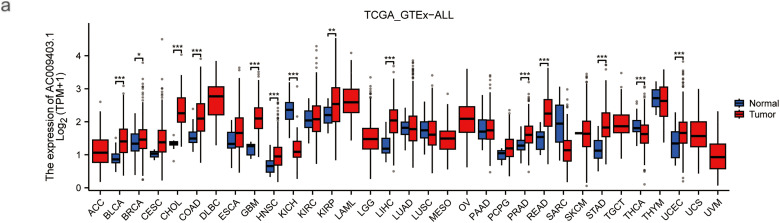

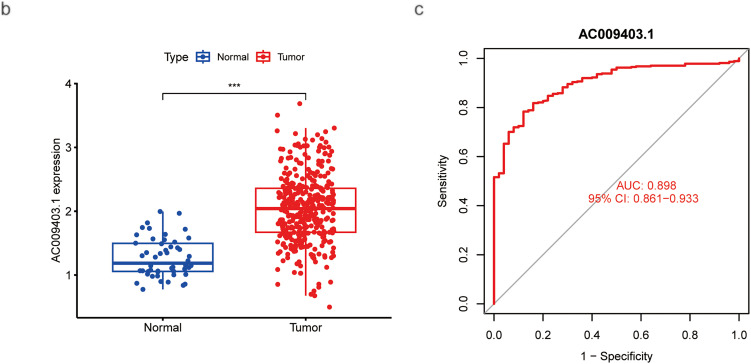
Evaluation based on AC009403.1 expression. (**a**) Expression and activity of AC009403.1 across various cancer types. (**b**) Expression of AC009403.1 in tumor vs. normal samples. (**c**) Sensitivity and specificity of ROC curves based on AC009403.1 expression (*p < 0.05; **p < 0.01; ***p < 0.001)

As shown in the pan-cancer analysis (Fig. 7a), AC009403.1 was highly expressed in multiple cancer types, although it showed minimal expression in certain tumors such as Kidney Chromophobe (KICH) and Thyroid carcinoma (THCA). Its expression was significantly elevated in HCC tissues relative to normal controls (*p* < 0.001; Fig. 7b). Furthermore, AC009403.1 demonstrated strong diagnostic performance for distinguishing HCC from non-cancerous tissues, achieving an AUC of 0.898 (95% CI: 0.861–0.933) in the ROC analysis (Fig. 7c).

**Figure 8:**
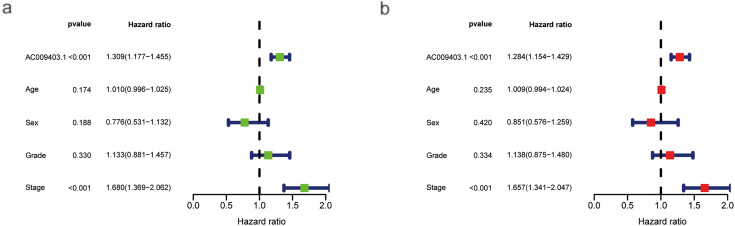

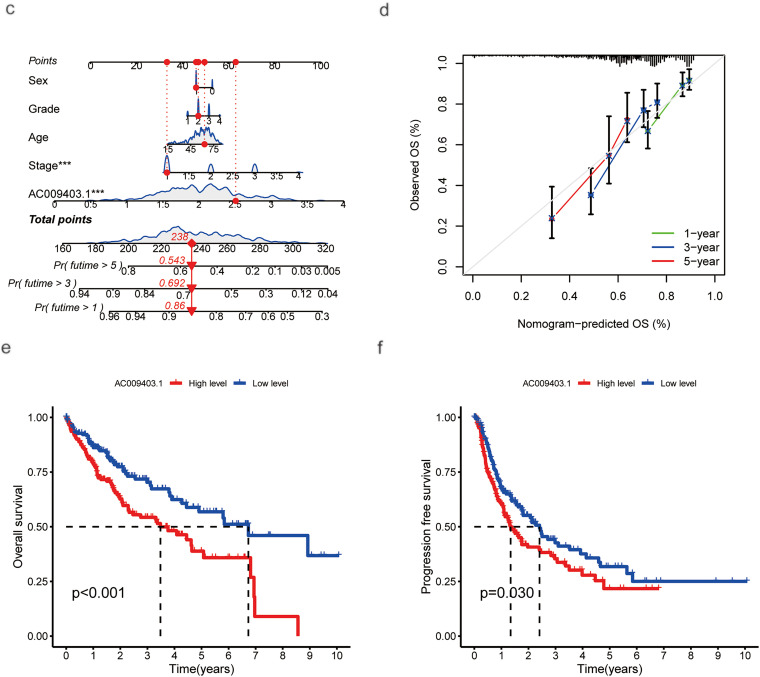
COX regressions, nomograms, and kaplan-meier curves based on AC009403.1 expression. (**a**) Univariate Cox regression analysis of the effects of clinical characteristics and AC009403.1 on HCC prognosis. (**b**) Multivariate Cox regression analysis of the effects of clinical characteristics and AC009403.1 on HCC prognosis. (**c**) Prediction of OS in HCC patients based on clinical characteristics and AC009403.1 expression using a nomogram. (**d**) Calibration curve assessing the accuracy of the nomogram. (**e**,**f**) Kaplan-Meier survival curves for HCC patients based on varying AC009403.1 expression (***p < 0.001)

Univariate Cox regression analysis identified advanced stage (HR = 1.680, 95% CI: 1.369–2.062) and high AC009403.1 expression (HR = 1.309, 95% CI: 1.177–1.455) as significant risk factors associated with poorer survival (Fig. 8a). Multivariate analysis confirmed both factors remained independent predictors of adverse outcomes (Fig. 8b). With AC009403.1 serving as a standalone prognostic indicator (HR = 1.284, 95% CI: 1.154–1.429). A prognostic nomogram was developed incorporating sex, grade, age, stage, and AC009403.1 expression levels to predict 1-, 3-, and 5-year overall survival probability (Fig. 8c). The calibration curves demonstrated close agreement between nomogram-predicted and observed survival rates at these time points (Fig. 8d). Kaplan-Meier analysis further indicated that patients with low AC009403.1 expression had significantly longer OS (Fig. 8e; *p* < 0.001) and PFS (Fig. 8f; *p* < 0.05) than those in the high-expression group.

**Figure 9:**
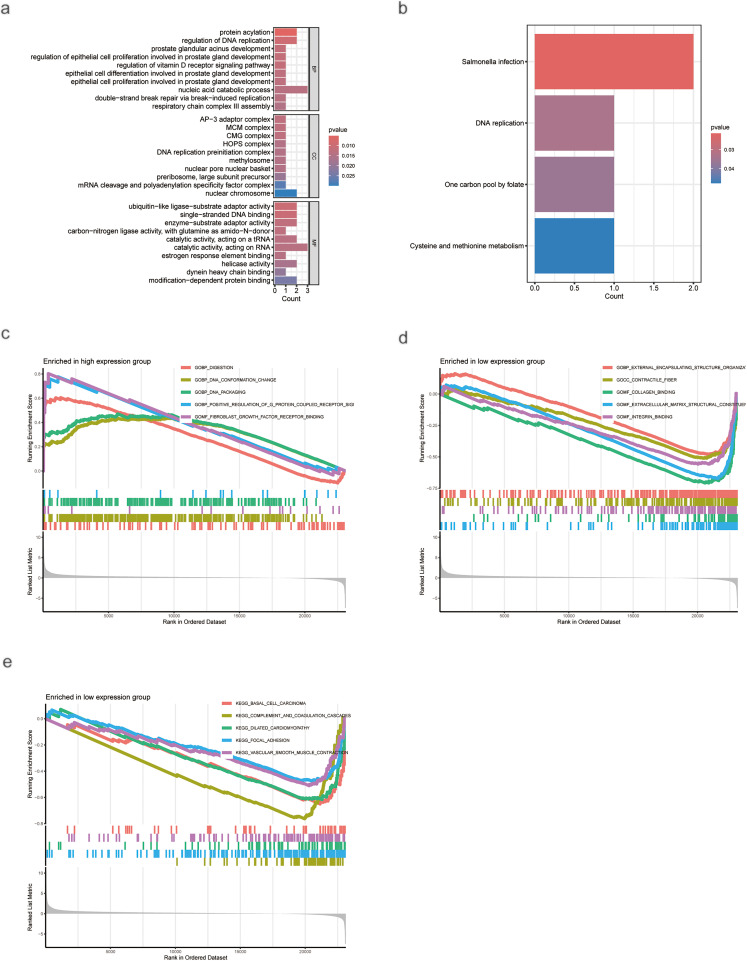
Enrichment analysis of AC009403.1 DEGs. (**a**) GO analysis of AC009403.1 DEGs. (**b**) KEGG analysis of AC009403.1 DEGs. (**c**–**e**) GSEA analysis of AC009403.1 DEGs

GO and KEGG enrichment analyses indicated that AC009403.1 was primarily associated with biological processes including nucleic acid catabolism, catalytic activity on RNA, defense responses to Salmonella infection, and the humoral immune response (Fig. 9a,b). GSEA based on GO terms showed that the high-expression subgroup was predominantly enriched in cell signaling and DNA structural changes (Fig. 9c), including pathways such as fibroblast growth factor receptor binding and G-protein-coupled receptor signaling. In contrast, the low-expression subgroup was enriched in terms related to cellular structure and function, such as collagen binding, integrin binding, and contractile fiber activity (Fig. 9d). Furthermore, KEGG pathway analysis via GSEA showed that the low-expression subgroup was associated with inflammatory responses and immune regulation (Fig. 9e), including complement activation, focal adhesion, and vascular smooth muscle contraction.

**Figure 10:**
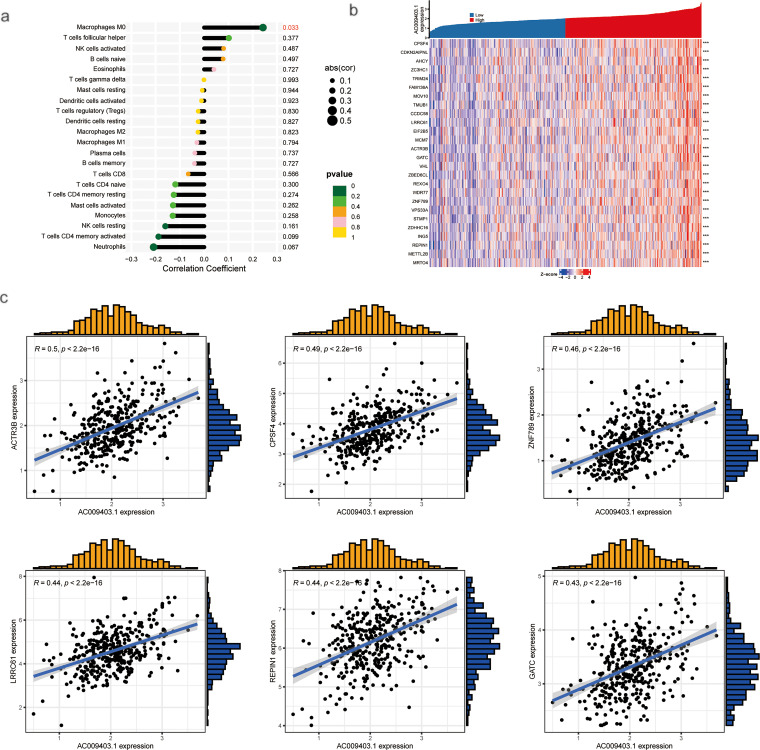
Correlations between AC009403.1 expression and immune infiltration, DEG analysis, and co-expression gene analysis. (**a**) Lollipop plot showing correlations between AC009403.1 and immune cells. (**b**) Heatmap of 27 DEGs with high and low expression of AC009403.1. (**c**) Correlation between AC009403.1 expression and genes (***p < 0.001)

AC009403.1 expression showed significant positive correlations with the infiltration levels of several immune cells, including M0 macrophages, T follicular helper cells, activated NK cells, naïve B cells, and eosinophils. Conversely, significant negative correlations were observed with neutrophils, activated CD4^+^ memory T cells, and resting NK cells (Fig. 10a). Co-expression analysis in HCC tissues identified 27 genes significantly correlated with AC009403.1 expression (|cor| > 0.4, *p* < 0.001). A heatmap visualizing these correlation patterns is presented in Fig. 10b. Among the most strongly correlated genes were ACTR3B, CPSF4, ZNF789, LRRC61, and REPIN1 (Fig. 10c).

**Figure 11:**
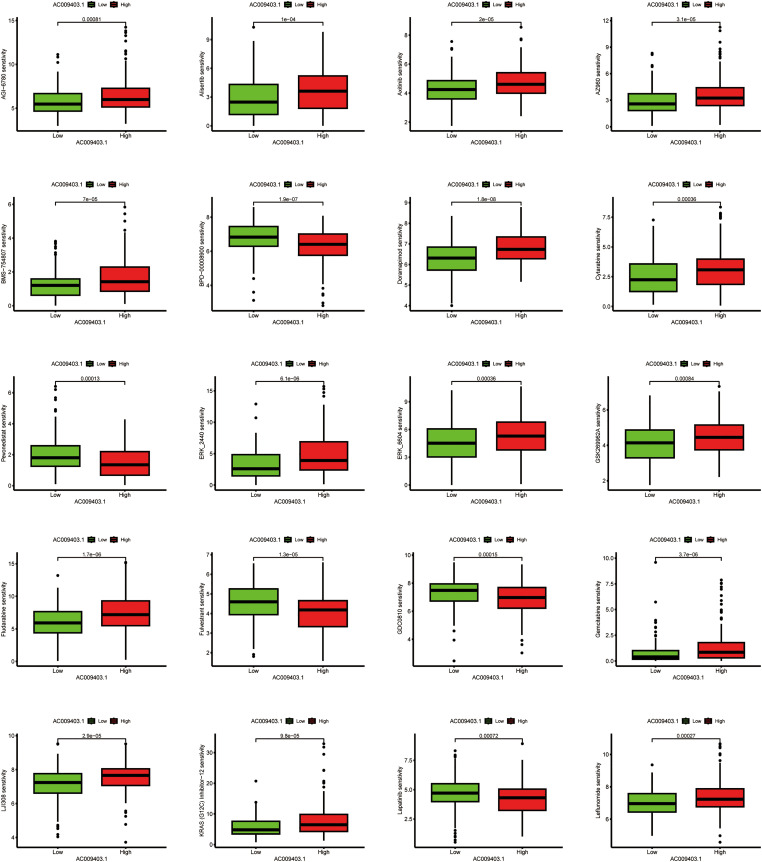
Associations between drug sensitivity and AC009403.1 expression. The exact *p*-value from the Wilcoxon rank-sum test is shown on the plot

To investigate potential therapeutic implications, we computationally estimated drug sensitivity based on AC009403.1 expression levels. The low-expression subgroup exhibited a potential increased sensitivity to agents such as Cytarabine, Irinotecan, Sorafenib, and others. In contrast, the high-expression subgroup exhibited a potential reduced sensitivity to drugs such as Fulvestrant, GDC0810, and Lapatinib (Fig. 11).

**Figure 12:**
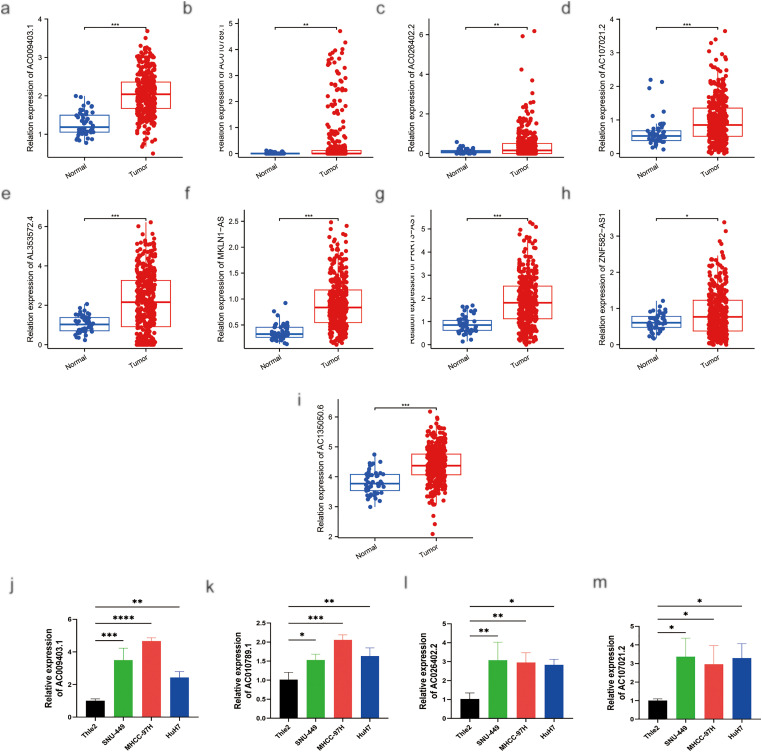

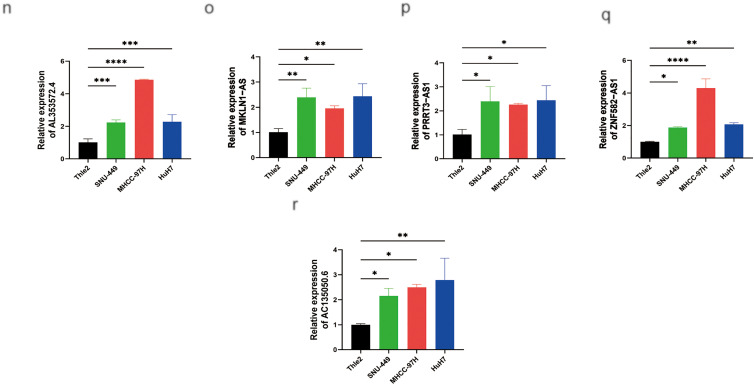
MRNA expression levels of nine genes by RT-qPCR. (**a**) Relative expression of AC009403.1; (**b**) AC010789.1; (**c**) AC026402.2; (**d**) AC107021.2; (**e**) AL353572.4; (**f**) MKLN1-AS; (**g**) PRRT3-AS1; (**h**) ZNF582-AS1; (**i**) AC135050.6 in HCC and healthy samples from TCGA databases. (**j**–**r**) RT-qPCR analysis of AC009403.1, AC010789.1, AC026402.2, AC107021.2, AL353572.4, MKLN1-AS, PRRT3-AS1, ZNF582-AS1, and AC135050.6 in THLE-2 cells and three HCC cell lines (HuH7, SNU-449, MHCC-97H). *p < 0. 05; **p < 0. 01; ***p < 0. 001; ****p < 0. 0001

As shown in Fig. 12a–i, the expression levels of nine palmitoylation-related lncRNAs (AC009403.1, AC010789.1, AC026402.2, AC107021.2, AC135050.6, AL353572.4, MKLN1-AS, PRRT3-AS1, and ZNF582-AS1) were analyzed in liver tissues using data from TCGA database. These lncRNAs were significantly upregulated in HCC tissues compared to normal liver tissues (tumor: *n* = 374; normal: *n* = 50). Furthermore, RT-qPCR analysis confirmed the elevated expression of these lncRNAs in HCC cell lines (HuH7, SNU-449, and MHCC-97H) relative to the normal human hepatocyte cell line THLE-2 (Fig. 12j–r).

## 4 Discussion

HCC is a prevalent malignancy characterized by high mortality and poor prognosis, despite the availability of various treatment options [2]. Therefore, reliable prognostic assessments and the identification of effective biomarkers are essential. In this study, we developed a comprehensive prognostic model based on nine palmitoylation-associated lncRNAs, which effectively stratified HCC patients into distinct risk categories with significant differences in clinical outcomes, molecular characteristics, and therapeutic responses. Several key findings merit emphasis.

First, we constructed a robust risk-scoring system centered on nine palmitoylation-related lncRNAs. This model differs from previous prognostic models in that it combines the features of lncRNA and palmitoylation to predict the prognosis of HCC. Regarding lncRNAs, prior studies have demonstrated their prognostic value across various cancer types, including rectal cancer, breast cancer, and lung cancer [31,32]. In HCC, lncRNAs such as HULC and MALAT1 have been shown to regulate macrophage polarization within the tumor microenvironment by modulating cytokine secretion and immune regulators, including IL-10 and TGF-β, thereby promoting an M2-like, pro-tumor phenotype that accelerates tumor progression [33]. Moreover, lncRNA-based prognostic signatures have been increasingly developed for HCC. For instance, a set of seven iron death-associated lncRNAs (e.g., LINC00942, AC131009.1, POLH-AS1, AC090772.3, MKLN1-AS, AC009403.1, and AL031985.3) has been established for predicting prognosis in HBV-related HCC [34]. Another six cuproptosis-related lncRNAs (AC026412.3, AC125437.1, AL353572.4, MKLN1-AS, TMCC1-AS1, and SLC6A1-AS1) have shown promise in predicting both survival and immunotherapy response [35]. It is noteworthy that several lncRNAs in our model, including MKLN1-AS, also appear in these previously reported signatures. Emerging evidence indicates that palmitoylation significantly influences ferroptosis [36], leading us to hypothesize that lncRNAs such as MKLN1-AS may indirectly modulate processes like ferroptosis or cuproptosis by regulating the stability or function of palmitoylated proteins. Furthermore, other lncRNAs in our signature, such as POLH-AS1, have been implicated in liver cancer progression and immune modulation [37], and ZNF582-AS1 may act as a tumor suppressor in breast cancer [38], supporting the biological relevance of our model.

Regarding palmitoylation, this reversible post-translational modification plays a crucial role in the development of tumors. Targeting palmitoylation or the enzymes responsible for palmitoylation (palmitoyl S-acyltransferases) is emerging as a promising therapeutic strategy for specific cancers [39]. For example, elevated PCSK9 expression promotes HCC cell proliferation and Sorafenib resistance, and inhibition of PCSK9 palmitoylation has been shown to enhance Sorafenib’s antitumor efficacy [40]. Additionally, palmitoylation of PD-L1 in the cytoplasm stabilizes the protein and impairs T cell-mediated antitumor immunity; blocking this modification enhances immune responses [41]. Although numerous prognostic models for HCC have been developed—such as those based on ferroptosis-, cuproptosis-, or sulfide-associated lncRNAs—these are primarily centered on specific modes of cell death. In contrast, our model focuses on palmitoylation, a fundamental regulatory mechanism that influences diverse oncogenic pathways. This fundamental difference in biological mechanism implies that our model may capture broader and more versatile oncogenic pathways. To the best of our knowledge, this is the first study to develop a prognostic model based on palmitoylation-associated lncRNAs in HCC, offering a novel perspective for understanding lncRNA-mediated regulatory mechanisms in this malignancy.

Second, HCC patients were stratified by risk score. Significant differences were observed between the high- and low-risk groups in terms of clinicopathological features, mutations, prognosis, TMB, and drug sensitivity [42,43]. High-risk patients exhibited shorter OS and PFS compared to low-risk patients. Additionally, high-risk individuals showed a significantly higher frequency of somatic mutations, which correlated with poorer prognosis and unfavorable outcomes. It has been established that a negative correlation exists between TMB and tumor prognosis, and high-risk patients with elevated TMB levels tend to have worse outcomes. In our study, the TP53 gene was the most frequently mutated in both patient groups. Accumulating evidence suggests that TP53 mutations may interact with lncRNA expression and palmitoylation pathways. For instance, lncRNAs have been implicated in modulating TP53 mutant functions [44]. Notably, a recent seminal study by Lin et al. reveals that mutant p53 itself is a key beneficiary of palmitoylation. They demonstrated that the lipogenic enzyme FASN promotes the palmitoylation, stabilization, and gain-of-function of mutant p53, thereby directly linking lipid metabolism and PTM to mutant p53-driven oncogenesis [45]. We hypothesize that this mechanism establishes a permissive microenvironment that enhances the oncogenic functions of the identified lncRNAs via aberrant palmitoylation. Validation of this hypothesis will be an important focus of future research. Computational analysis of drug sensitivity suggested that low-risk patients may be more sensitive to Cytarabine, Irinotecan, Sorafenib, Olaparib, Oxaliplatin, Fludarabine, Tamoxifen, and KRAS (G12C) Inhibitor-12. In contrast, high-risk patients showed increased sensitivity to 5-Fluorouracil, Foretinib, Fulvestrant, and Alpelisib. Notably, several of these predicted agents (e.g., Sorafenib, Oxaliplatin, and 5-Fluorouracil) are consistent with current standard treatments for HCC [3]. These results highlight the potential of our risk-scoring system to inform therapeutic decision-making and contribute to the development of personalized treatment strategies for patients with HCC.

Third, GSEA and GO enrichment analyses suggest that the low-risk group was significantly enriched in immune-related processes, such as immunoglobulin complex binding, antigen binding, immunoglobulin production, and B cell-mediated immunity. Furthermore, this group was also predominantly associated with pathways regulating cellular metabolic signaling, including primary bile acid biosynthesis, fatty acid metabolism, and amino acid metabolism. In contrast, the high-risk group showed strong involvement in cell cycle-related signaling, including DNA replication and ribosome biogenesis. These findings emphasize the biological significance of palmitoylation-related lncRNAs in immune regulation, cell cycle progression, and metabolism.

Fourth, immune correlation analysis demonstrated both positive and negative correlations between risk scores and various immune cell types. As immunotherapy has become an important treatment option for HCC [46,47], our study further supports previous findings that tumor-infiltrating lymphocytes play a critical role in immune suppression during HCC progression. Various immune cells, including neutrophils, monocytes, hepatic lymphocytes (B cells, CD8^+^ T cells, and CD4^+^ T cells), and tumor-associated macrophages, contribute to the tumor immune response [48–50]. New research suggests that chemotherapy can enhance T cell-mediated cytotoxicity by stimulating common lymphoid progenitor cells and enhancing CD8^+^ T cell-dependent tumor cell targeting [51]. The complexity of the tumor immune microenvironment is increasingly recognized; recent advances in single-cell RNA sequencing (scRNA-seq) have greatly refined our understanding of cellular heterogeneity and immune evasion mechanisms in HBV-HCC, offering new insights into its pathogenesis and treatment resistance [52]. In addition, our findings indicated a positive correlation between risk scores and immune cells such as T follicular helper cells, activated NK cells, M0 and M1 macrophages, and common lymphoid progenitor cells, and an inverse correlation with activated mast cells, granulocyte-monocyte progenitors, neutrophils, and resting memory CD4^+^ T cells. These results suggest that risk scores may reflect intricate interplay within the immune microenvironment, potentially informing immunotherapeutic strategies.

A particularly novel aspect of our study is the identification and preliminary characterization of AC009403.1, a previously uncharacterized lncRNA in the context of HCC. Analysis of TCGA data revealed significant overexpression of AC009403.1 in HCC tumors compared with normal tissues. Multivariate Cox regression and nomogram analyses confirmed that high AC009403.1 expression serves as an independent prognostic factor associated with poorer OS and PFS. Functional enrichment analyses suggested that lower expression of AC009403.1 was primarily linked to immune regulatory processes. Co-expression analysis identified 27 genes positively correlated with AC009403.1 expression, including ACTR3B, CPSF4, ZNF789, LRRC61, and REPIN1, providing further insights into potential molecular pathways. Additionally, drug sensitivity analysis indicated that patients with low AC009403.1 expression showed increased responsiveness to several commonly used HCC treatments, highlighting its potential as a therapeutic target. Based on these findings, we hypothesize that AC009403.1 may possess a potential mechanistic role in palmitoylation, which we shall explore in greater depth in future investigations.

Despite these promising results, the study has some limitations. First, although RT-qPCR was performed for partial validation, our results are largely derived from bioinformatic analyses; thus, more extensive functional experiments, both *in vitro* and *in vivo*, are necessary to establish causal relationships. Second, the retrospective nature and exclusive reliance on TCGA database introduce specific biases. The TCGA-LIHC cohort predominantly comprises Western patients, which may limit the generalizability of our model to global populations where other etiologies (e.g., HBV) are prevalent. Notably, the absence of etiology-specific stratification, such as comparing HBV/HCV-related HCC with NAFLD-related HCC, precludes conclusions regarding whether the prognostic performance of our signature is consistent across molecularly distinct HCC subtypes. Third, clinical validation is currently lacking, and the translational applicability of our model requires further verification in prospective, multi-ethnic cohorts with well-annotated etiological information. Finally, the relatively small sample sizes in the training and validation sets may affect the stability and generalizability of the model. Therefore, larger multi-center studies and comprehensive experimental investigations are essential to validate, refine, and ultimately translate our prognostic signature toward clinical utility.

## 5 Conclusion

In conclusion, we have developed a novel prognostic model based on nine palmitoylation-associated lncRNAs for HCC. Our findings suggest that this signature may predict survival, tumor microenvironment composition, and response to immunotherapy in patients with HCC. These results highlight its potential as a prognostic tool to inform therapeutic decisions. To facilitate clinical translation, our future work will proceed through three critical steps: retrospective validation in multi-ethnic cohorts, prospective multi-center clinical studies, and ultimately, the development of a standardized clinical assay. These efforts are essential to translate this signature from a computational model into a tool for personalized HCC management.

## Supplementary Materials



## Data Availability

Data available on request from the authors.

## Supplementary Materials

The supplementary material is available online at https://www.techscience.com/doi/10.32604/or.2025.070567/s1.
